# Fiber Bragg Grating Sensor to Monitor Stress Kinetics in Drying Process of Commercial Latex Paints

**DOI:** 10.3390/s100504761

**Published:** 2010-05-11

**Authors:** Ivo de Lourenço, Gustavo R. C. Possetti, Marcia Muller, José L. Fabris

**Affiliations:** Programa de Pós-Graduação em Engenharia Elétrica e Informática Industrial, Universidade Tecnológica Federal do Paraná, 80930-901 Curitiba, Brazil; E-Mails: lourenco@utfpr.edu.br (I.L.J.); gustavo_possetti@yahoo.com.br (G.R.C.P.); mmuller@utfpr.edu.br (M.M.)

**Keywords:** fiber Bragg grating, optical fiber strain sensor, paint drying process

## Abstract

In this paper, we report a study about the application of packaged fiber Bragg gratings used as strain sensors to monitor the stress kinetics during the drying process of commercial latex paints. Three stages of drying with distinct mechanical deformation and temporal behaviors were identified for the samples, with mechanical deformation from 15 μm to 21 μm in the longitudinal film dimension on time intervals from 370 to 600 minutes. Drying time tests based on human sense technique described by the Brazilian Technical Standards NBR 9558 were also done. The results obtained shows that human sense technique has a limited perception of the drying process and that the optical measurement system proposed can be used to characterize correctly the dry-through stage of paint. The influence of solvent (water) addition in the drying process was also investigated. The paint was diluted with four parts paint and one part water (80% paint), and one part paint and one part water (50% paint). It was observed that the increase of the water ratio mixed into the paint decreases both the mechanical deformation magnitude and the paint dry-through time. Contraction of 5.2 μm and 10.4 μm were measured for concentrations of 50% and 80% of paint in the mixture, respectively. For both diluted paints the dry-through time was approximately 170 minutes less than undiluted paint. The optical technique proposed in this work can contribute to the development of new standards to specify the drying time of paint coatings.

## 1. Introduction

Nowadays optical fiber sensors have been widely applied in many areas of science and technology. These sensors are able to probe a variety of measurands and, therefore, can be used in many different sectors, such as industrial, military, aerospace, chemistry, petrochemical, telecommunication and medicine, for different applications [1–6].

Fiber Bragg grating (FBG) sensors have been exploited as intrinsic optical fiber sensors to measure strain with superior characteristics such as relatively small size, high sensitivity, inertness to electric or magnetic interference, punctual or quasi-distributed measurement ability associated with the wavelength multiplexing capability, and electrical passivity at the measurement point [7]. Also, as FBG are intrinsic optical fiber devices they can be easily integrated in an optical link, eliminating the need for electrical cabling common to electrical transducers. The main advantages of FBG over others optical sensors are its low cost, good linearity, wavelength multiplexing capability, resistance to harsh environments and transduction mechanism, which eliminates the need for referencing as in interferometric sensors [8]. These features have made FBG sensors very attractive for quality control during construction, health monitoring after building and impact monitoring of large composite or concrete structures [9–11]. FBG strain sensors seem to be ideal to realize the monitoring of smart structures where optical fiber sensors are embedded in the structure [12,13].

The paint industry offers a wide range of quality control testing of its products. Careful monitoring and process control are needed to obtain and adjust the operating parameters of the system. However, the major problem is that the painting process has both mechanical and chemical elements that must be controlled. Besides, the measurement techniques available for this aim are incipient and critically dependent on the subjective human sense.

In Brazil, for example, the paint industry employs direct contact methods that are influenced by the operator sense to check the drying time of its formulations. The determination of the drying steps described by the Brazilian Technical Standards (NBR 9558) is made by the contact of the fingers and fingernails, and often leads to a lack of reproducibility and repeatability [14]. In this determination method, the drying process is sub-divided into four steps: dry-to-touch, tack-free, dry-to-handle and dry-through. However, for most commercial applications a non-contact method is required as the operator’s touch can damages the painted surface. Besides, personal impressions can introduce errors in the determination of the paint drying time.

Basically, the paint film drying corresponds to a change from liquid to solid state. As the paint dries on the substrate, a firmly bonded film is formed [15]. The properties of this film are determined both by the substrate and its pretreatment (cleaning, degreasing), as well as by the composition of the coating and the method of application used. For coatings to adhere, surfaces must be free from oily soils, corrosion products and loose particulates. The choice of cleaning method depends on the substrate, as well as the size and the shape of the object. Temperature, humidity and airflow are environmental factors that influence the drying times of paint films. The drying time is also dependent on the used solvents and binders [16].

Additionally, as the paint is applied, it will flow and stabilize into a relatively transparent and uniform film. This stage usually occurs by evaporation of volatile solvents from an organic solvent system or of water from a latex system. After the paint has stabilized, the drying process starts. During this time, the paint films are subjected to a great variety of mechanical forces and deformations, which are difficult to detect by methods available at present, especially by the standard test based on human sense.

The mechanism of film formation has been extensively studied in the past, however there are still many unresolved questions. An overview of polymer latex film formation and properties was compiled from the literature by Steward *et al.* [16]. Routh and Russel provide a review of the literature and discuss the proposed deformation mechanisms responsible for the particle deformation (wet sintering, dry or moist sintering, capillary deformation, receding water front and Sheetz deformation) [17]. Although the mechanisms responsible for the film formation are subject of controversy, the process is usually divided into three stages: (Stage I) evaporation of water followed by particle concentration and ordering, (Stage II) particle deformation, and (Stage III) polymer chain diffusion across particle boundaries. Despite the importance of the mechanical stress in the film formation, the use of experimental techniques to monitor the evolution of stress during the drying process is still incipient. Petersen *et al.* used a cantilever method to follow the macroscopic stress evolution, and information about the internal micromechanics processes were provided by the authors [18]. The films were dried on a flexible substrate, and the substrate curvature caused by the film stress evolution was detected with a laser beam reflected from a small mirror fixed to the cantilever. The authors associated the macroscopic stress observed during the film drying with the three stages usually described in the literature. According to the authors, the first stage in the film formation, corresponding to the water evaporation, occurs in a relatively short period of time. In this stage, the curvature of the liquid-air interface creates the capillary pressure, according to the Sheetz model [19], which compresses the film and is responsible for a dilatational lateral stress. The authors identify the time of maximum tensile stress measured by the cantilever with the end of the Stage II. The third stage, observed in the experiment as a stress relaxation, takes place after the maximum tensile stress. In this stage, the interdiffusion of the polymer chains occurs. As the skin at the film-air interface is responsible for most of the tensile stress, the drying process is influenced by the film thickness. The drying time is slower and the averaged stress is lower for larger film thickness.

In this work, a fiber Bragg grating sensor head specially designed to follow the macroscopic stress evolution during film formation of two different commercial latex paints is presented. The sensor working principle is based on the FBG sensitivity to longitudinal strain (the geometrical measure of deformation representing the relative displacement between particles in a material body). The influence of solvent concentration in the drying process is also investigated in samples obtained by the addition of distilled water to the paint.

## 2. Principle of Operation of the Optical Fiber Sensor

A fiber Bragg grating is a periodic modulation induced in the core refractive index of an optical fiber whose periodicity is in the range of micrometers. This periodic structure couples the optical power guided by core fundamental mode to core contra-propagating mode at a particular wavelength (*λ*_B_) called the Bragg wavelength, which fulfills the Bragg condition [20]:

(1) $$\lambda_{B}=2n_{\mathrm{eff}}\;\Lambda$$

Equation 1 indicates that the Bragg wavelength (*λ_B_*) depends on the effective refractive index of the core mode (*n_eff_*), besides of the grating periodicity (*Λ*).

External parameters can affect both *n_eff_* and *Λ* resulting in changes on the *λ_B_*. This dependency allows the FBG to be applied as a sensor of temperature and strain [21]. The strain response occurs because of both the physical elongation of the optical fiber (and the corresponding change in *Λ*), and the change in refractive index of the optical fiber due to elasto-optic effect (that consequently modifies *n_eff_*) [22]. The inherent thermal expansion of the optical fiber material (that changes *Λ*) and the dependence of the refractive index of the optical fiber with the temperature due to thermo-optic effect (that modifies *n_eff_*) justify the FBG temperature response [23]. Mathematically, the shift in the Bragg wavelength (*Δλ_B_*) due to an applied strain and temperature change is given by [24]:

(2) $$\Delta \lambda_{B}=2\left(\Lambda \frac{\partial n_{\mathrm{eff}}}{\partial l}+n_{\mathrm{eff}}\frac{\partial \Lambda }{\partial l}\right)\Delta l+2\left(\Lambda \frac{\partial n_{\mathrm{eff}}}{\partial T}+n_{\mathrm{eff}}\frac{\partial \Lambda }{\partial T}\right)\Delta T$$

where, *l* is the FBG length, and *T* is the temperature.

The Equation 2 shows that any change in wavelength, associated with the action of an external perturbation to the grating, is the sum of both strain and temperature terms. Therefore, in sensing applications where only one perturbation is of interest, the decoupling of either temperature effect or strain effect becomes necessary [25]. The simplest approach for elimination of cross-sensitivity problems is to use two FBG, with one of them isolated from the unwanted perturbation [26]. Typical values for FBG sensitivities with Bragg wavelength close to 1.5 μm are 1 pm/με and 10 pm/°C [27]. Hence, when the temperature effects are compensated and/or controlled, the FBG response can be associated only with strain changes. In this sense, the FBG can be used to measure the strain kinetics during the drying process of paints.

## 3. Methodology

To realize the experiments, six FBG (FBG1 to FBG6) were engraved in photosensitivity single-mode optical fiber and employed as optical transducers. Each FBG was photo-written with a KrF excimer laser (Coherent, Xantos XS), operating at 248 nm, using the direct phase-mask technique [28]. For writing the FBG, the optical fiber was exposed to UV laser beam, with 10 mJ/cm^2^ of fluency, and frequency of 200 Hz for approximately 10 minutes.

The FBG1, with Bragg wavelength of 1,546.53 nm at 20.0 °C, was selected only for temperature measurement. For this purpose, the FBG1 thermal response curve was determined using a Peltier cell monitored by type K thermocouple with resolution of 0.1 °C. The Bragg wavelength position was determined for the range between 0.0 °C and 50.0 °C in steps of 5.0 °C.

The others gratings (FBG2 to FBG6) were selected in order to perform the strain measurements. These FBG were individually packaged, producing the sensor heads used to measure mechanical deformation associated with the paint drying process. The FBG packaging has two main functions: allows a better measurement of the paint mechanical deformation inherent to the drying process and also avoids paint poor adhesion to the bare fiber.

**Figure 1.**
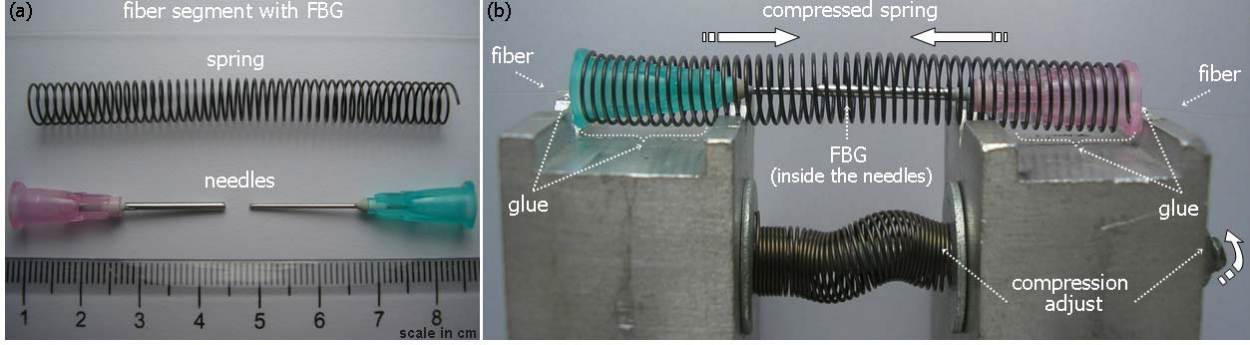
Sensor head construction: (a) elements used to build the sensor head, (b) assembled sensor head installed on the device for gluing and pre-straining the FBG.

For the FBG packaging, two metallic needles of injection syringes with diameters of 0.80 mm and 1.20 mm were used—one put inside the other—and placed inside a spring. The wire diameter of the spring is 0.45 mm, while the spring diameter is 7.00 mm, resulting in an elastic constant of 1.0 N/m. In Figure 1(a), the elements used to build the sensor head are shown in scale. In the next step, the fiber with the FBG was inserted into the needles; after that the whole set was adjusted to the device responsible for compressing the spring, Figure 1(b). Then, the optical fiber and the spring were glued with cyanoacrylate to the plastic extremities of the needles. After the drying, the spring is released, subjecting the FBG to an initial strain.

**Figure 2.**
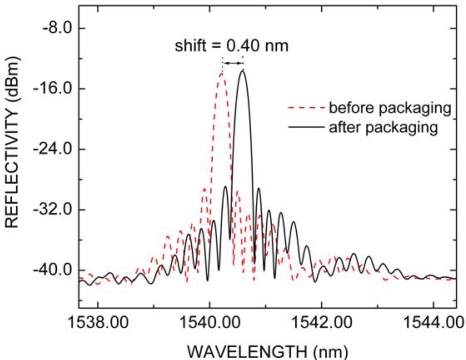
FBG2 reflection spectrum before (red dashed line) and after (black solid line) packaging.

After the FBG packaging, the initial longitudinal strain applied to the grating by the spring changes the characteristics of the FBG reflection spectrum inducing a red shift in Bragg wavelength, which is measured by the optical sensing interrogator (Micron Optics, sm125) with 5 pm of wavelength stability in wavelength range between 1,520 nm and 1,570 nm. The effect of packaging in FBG2 reflection spectrum can be seen in Figure 2. The maximum reflectivity, before (red dashed line) and after (black solid line) packaging, is approximately equal to 21.5 dB. However, the Bragg wavelength, for which the initial position is 1,540.18 nm at 20.0 °C, suffers red shift of about 0.4 nm after the FBG2 packaging. Similar behavior was also registered for the other FBGs packaged. The Bragg wavelength position (*λ_B_*), before and after packaging, and the shift (*δλ*) induced by packaging for all FBG employed are summarized in Table 1. The *δλ* value defines the dynamical range for the strain sensor.

To access the dynamics of paint drying, a thin layer of paint is deposited all over the spring that comprises the sensor head. Internal forces along the polymer occurring during the drying process, which result either from film contraction or expansion, are transmitted to the spring coils on to which the film is adhered. This contraction or expansion is consequently communicated to the fiber with the FBG. The overall effect is to produce a detectable strain on the sensor with a non-linear temporal behavior as the drying evolves, each specific rate of strain evolution being associated with the three described stages of paint drying normally presented in the literature (evaporation, particle deformation and polymer chains interdiffusion).

The initial strain applied to FBG is to guarantee that the grating always remains stretched during the paint drying process. This procedure assures that the fiber remains unbent even if the fiber suffers a contraction due to the drying process, within the pre-defined dynamic range. Despite the different values in initial strain applied to each packaged FBG, no significant change in the strain sensitivities occurred for all devices.

The packaged FBG2 was strain calibrated using a linear step motor translation stage electronically controlled and connected to a dial indicator (Mitutoyo 2046F) with 0.005 mm of resolution. The segment of optical fiber with known size that contains the grating was fixed to displacement stages and the fiber was stretched. In this configuration, the resolution of calibration strain system is 100 με (1 με equals to a deformation of 1 μm in a total length of 1 m). The Bragg wavelength position was determined for the range between 0 με and 1,000 με in steps of 200 με. The monitoring of paint stress kinetics during its drying is a destructive experiment. Then, gratings FBG3 to FBG6 were selected to realize these particular experiments and the FBG2 was only employed for strain calibration purpose.

**Figure 3.**
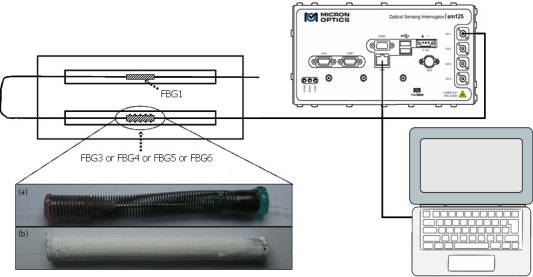
Schematic of experimental set-up used to monitor the paint stress kinetics during its drying.

Figure 3 shows a schematic diagram of experimental set-up used to measure the strain associated with the mechanical stress kinetics during the drying process of paints. In this apparatus, two FBG were connected in series and coupled to optical sensing interrogator (Micron Optics, sm125). The interrogator was connected to a notebook via Ethernet protocol and permitted the reading and the storage of FBG reflection spectra and their respective Bragg wavelength positions. The FBG1 was left on a planar surface, free of any strain influence, and used only to measure temperature changes. Therefore, the FBG1 wavelength response was used, in all experiments, to correct the thermal effects presents in packaged FBG responses. To minimize temperature changes and to avoid influence of air currents, the set-up was isolated from the external ambient by a transparent plastic box with dimensions of 30 × 35 × 35 cm. For all experiments, the temperature and the relative humidity of ambient isolated were measured by SHT75 digital humidity and temperature sensor as summarized in Table 2.

In the experimental set-up, one sensor head was connected in series with FBG1 as showed in Figure 3, and used to monitor the strain during the drying process of two different commercial latex paints (Paint1 and Paint2). As the experiment is a destructive test, the sensor head comprised by the packaged FBG3, FBG4, FBG5 or FBG6 needs to be replaced after each measurement. Each presented result for FBG3 to FBG6 is representative of a set of three repetitions with different sensor heads, with a fluctuation in the Bragg wavelength shift lower than 12%. This fluctuation, besides establishing a confidence level for the measurement in the film deformation, indicates if the sensor is properly working, as they were not individually strain-calibrated. Basically, the compositions of paints chosen for experiments were water emulsion snow white latex paint, which mainly contained: acrylic resins, pigments, coalescent, bacteria resistance (Paint1) and acrylic and vinyl resins, pigments, additives, bacteria resistance (Paint2). The overall composition of the final product is unknown as it is industrial secret information.

The spring of each packaged FBG was completely covered with a thin layer of latex paint using a paintbrush. The drying process was monitored by measuring wavelength shifts of gratings at regular intervals of one minute. In parallel, subjective human tests of drying were carried out under the suitable conditions described in the Brazilian Technical Standard NBR 9558 [14]. To avoid the influence of the finger contact with the latex in the sensor response, these qualitative steps were identified by using a second spring with the same characteristics of springs employed in the packaged FBG and completely covered with a thin layer of latex paint.

At each regular time interval of 10 minutes, the paint on the second spring was touched with a clean and degreased finger and the sensation interpreted according to the four steps of NBR 9558: dry-to-touch, tack-free, dry-to-handle and dry-through.

For experiments with dilute samples, a proper amount of solvent was added to the Paint2 according to the manufacturer’s instruction. The drying process was monitored for a dilute latex paint solution of four parts paint and one part distilled water (80% paint) and one part paint and one part distilled water (50% paint). The paint manufacturer recommends the addition of water up to 50% for the first coating. After this dilution proportion, the diluted paint will not form a thick dry film.

## 4. Results and Discussion

**Figure 4.**
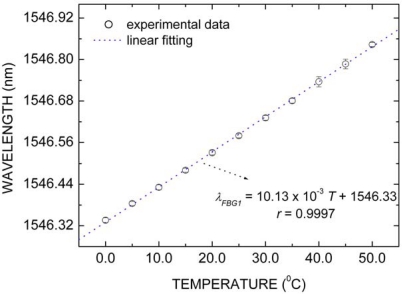
FBG1 response (*λ_FBG1_*) in terms of temperature (*T*) changes.

Figure 4 shows the FBG1 response (λ*_FBG1_*) in terms only of temperature (T) changes. The temperature increasing caused linear red shifts in Bragg wavelength. The blue dotted curve represents the linear fitting to experimental data, for which correlation coefficient (*r*) is approximately 1. The slope of the curve is the FBG1 thermal sensitivity, which is 10.13 ± 0.14 pm/°C. Type A (random) and type B (systematic) uncertainties were considered in the estimative of combined uncertainty [29]. Each experimental point in Figure 4 was determined by three independent measurements, the experimental standard deviation of the mean representing the Type A uncertainty. For Type B uncertainty, two components were considered: 5 pm of wavelength stability for the sm125 optical interrogator and 0.1 °C for the thermocouple resolution, transformed to wavelength uncertainty by using an empirical first-order Taylor series expansion based on the estimated sensitivity coefficients. For both components, a rectangular probability distribution was assumed. The final temperature uncertainty (±0.14 pm/°C) was obtained by adjusting a linear function to the experimental data of Figure 4, using errors as weight. All FBG used in this work were fabricated with the same photo-writing parameters (optical fiber type, fluence per laser pulse, *etc*.) and present similar spectral characteristics. For these reasons, we expect quite similar temperature sensitivities for all FBG. The wavelength shifts due to only temperature effects that occurred in any packaged FBG employed as strain sensor were compensated by subtracting its wavelength response from FBG1 response. Furthermore, in any experiment, the calibrated FBG1 can be used to measure the paint drying absolute temperature.

**Figure 5.**
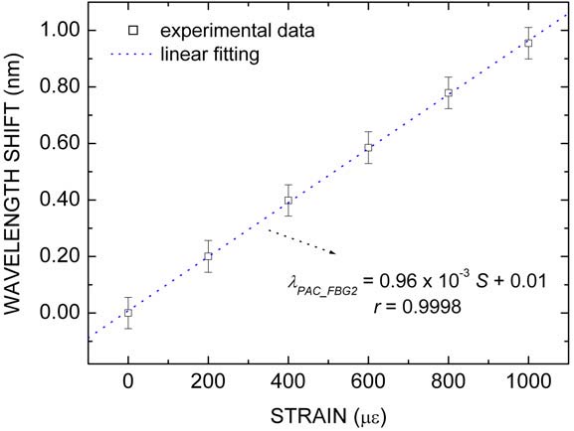
Packaged FBG2 wavelength shift (*λ_PAC_FBG2_*) in terms of strain (*S*) changes.

Figure 5 depicts the packaged FBG2 wavelength shift (*λ_PAC_FBG2_*) in terms only of strain (*S*) changes. A linear relationship between the sensor wavelength shift and the strain variation is also observed. From the linear fitting to experimental data, a strain sensitivity of 0.96 ± 0.07 pm/με was obtained. The same procedure used to estimate the temperature uncertainty in Figure 4 was employed to estimate the combined uncertainty in the strain sensitivity in Figure 5. In this case, for Type B uncertainty, the linear translation stage resolution was considered to be 100 με. As was justified and described above, we also expect quite similar strain sensitivity for all packaged FBG.

**Figure 6.**
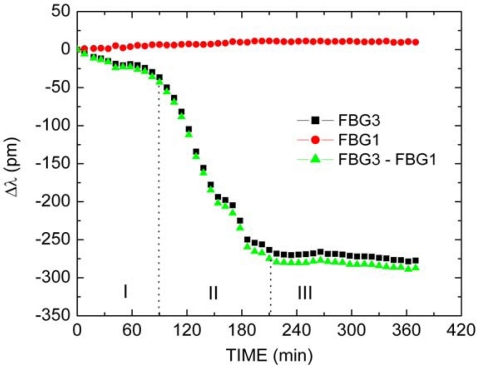
FBG1, packaged FBG3 and thermal compensated FBG3 responses as a function of time during the Paint1 drying process.

**Figure 7.**
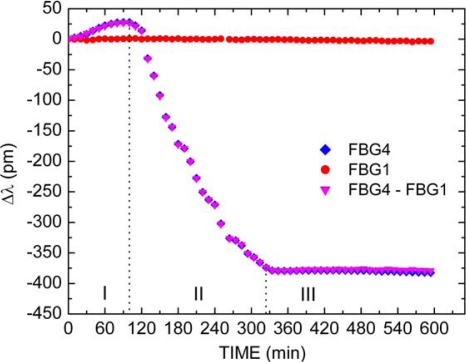
FBG1, packaged FBG4 and thermal compensated FBG4 responses as function of time during the Paint2 drying process.

**Figure 8.**
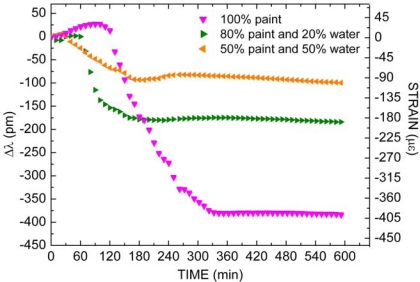
Packaged FBG4, FBG5 and FBG6 responses as function of time during the Paint2 drying process mixed with different water concentration.

The FBG1 and packaged FBG3 responses as function of time during the Paint1 drying process are presented in Figure 6. The values of Bragg wavelength of each FBG were subtracted from the respective position at the start of the experiment. As previously mentioned, the FBG1 was only subjected to temperature changes and during the experiment a shift of about 15 pm was observed ( 
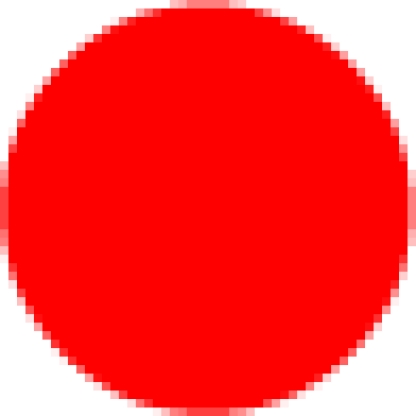
), relating to a temperature change of approximately 1.5 °C. The packaged FBG3 response (■) shows a different behavior due to the combined effects of temperature and mechanical deformation induced by the paint drying process. The relative wavelength from packaged FBG3 response minus relative wavelength from FBG1 response extracts the thermal effect in packaged FBG3 response, and shows only the strain effect due to spring contraction caused by paint film contraction ( 
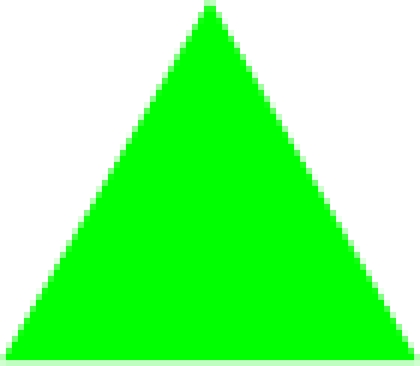
). The spectral behavior of packaged sensor for temperature variations was compared with the spectral behavior of loose FBG1, as well as with the spectral behavior of packaged sensor with a layer of dry paint, under the same temperature changes (∼1.5 C). This procedure allows accessing the influence of the materials with different thermal expansion coefficients involved in the experiment in the thermally-induced Bragg wavelength shifts. For the three situations, the same temporal pattern was observed, however with differences in the wavelength shifts magnitudes. The maximum difference in the wavelength shift observed between packaged and loose FBG was ∼16 pm, this difference decreased to ∼5 pm when the paint applied to the sensor becomes dry. Although the observed thermal sensitivities are different, the magnitude of such observed wavelength shifts are small when compared with the magnitude of wavelength shifts observed as the drying process evolves (∼300 pm). Consequently, under the temperature controlled conditions employed during the experiments, the thermal behavior of loose FBG1 can be used to provide small corrections due to temperature fluctuations. Data in Figures 6, 7 and 8 are presented in time intervals of about 10 minutes just to make the graphs clear, in spite of the fact that they were measured in time intervals of 1 minute.

To assure that the Bragg wavelength shifts observed are not associated with changes in temperature due to possible endothermic/exothermic reactions from the paint drying process, the temperature of a thermocouple, covered with a layer of paint under the same experimental conditions present along the experiment with the sensor head, was monitored. Just after the paint was applied with the brush, a decrease in the temperature of about 1.5 °C within a time interval of 30 s was verified. Within the next five minutes, the temperature reached the environment temperature. Therefore, possible endothermic/exothermic reactions affect the temperature only in the very beginning of the temporal scale of the experiment, which allows assigning the observed Bragg wavelength shifts mainly to the physical contraction/expansion of the paint samples.

The compensated FBG3 response shows that during the Paint1 drying process there are, mainly, contraction effects. Three stages (I, II, and III) with distinct temporal behaviors can be identified during the drying process. In the Stage I, which occurs during approximately the first 80 minutes, a total wavelength shift of approximately 35 pm is observed. In this stage, the paint contraction is probably associated with water evaporation. This increases the paint density until the particles begin to get in contact with each other. In the Stage II, which lasts 130 minutes, a stronger and more complex mechanical deformation is observed, resulting in a total wavelength shift of approximately 245 pm. In this stage, stresses in the film deform the particles leading them into irreversible contact. The process of water evaporation remains, but the overall rate of evaporation decreases during this stage. Finally, in the Stage III, which initiates after 210 minutes from the beginning of the experiment, there is a total shift of 10 pm and, therefore, a less significant mechanical deformation takes place. During this stage, there is an inter-diffusion of polymer chains and the film develops its final characteristics. It is observed that at the end of drying process, the total FBG3 wavelength suffers blue shift of 290 pm, corresponding to a decrease of approximately 302 με in the initial strain applied to the grating by the spring, which relates to a final contraction of about 15 μm in the longitudinal film dimension (or equivalently 0.03%). No cracking was observed in the film after the 370 minutes. As the paint thickness will affect the coating drying time, non-uniformities along the paint layer on the sensor head can lead to different localized drying behaviors along the spring. However, as the experiment supplies an averaged measurement along the entire spring, such non-uniformities in the paint thickness may appear solely as a secondary effect in the FBG spectrum.

The aforementioned tests of paint drying based on Brazilian Technical Standards were also investigated. The four steps: dry-to-touch, tack-free, dry-to-handle and dry-through were identified and occurs after approximately 60, 85, 90 and 95 minutes, respectively, after the beginning of the experiment for Paint1.

Figure 7 shows the FBG1 ( 
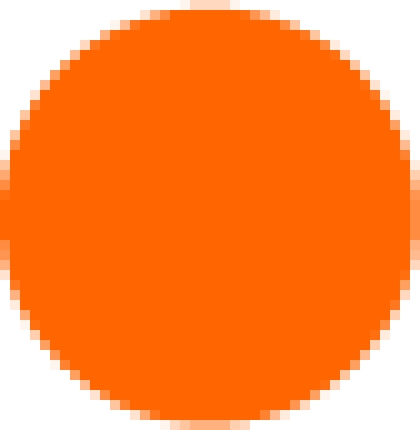
), the packaged FBG4 ( 
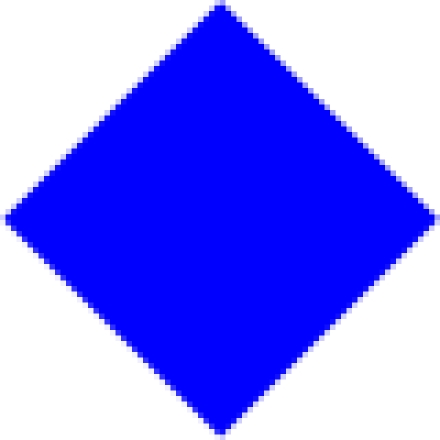
) and the thermal compensated FBG4 ( 
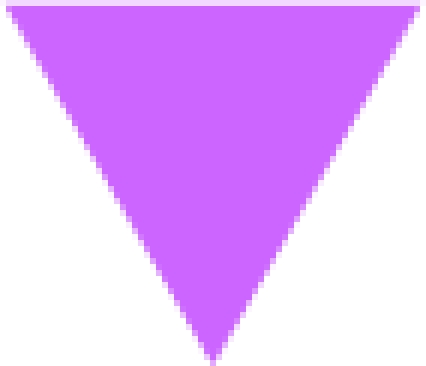
) responses as function of time during the Paint2 drying process. It should be noted that three stages with distinct temporal behaviors can also be identified in the drying process. Apart from the Stage I, both latex paints show a similar behavior during film formation. For the Paint2, however, Stage I is composed by expansion effects denoted by a red wavelength shift of approximately 26 pm during the first 100 minutes. This represents a deformation about 27 με of the spring, indicating an expansion of approximately 1.4 μm. The commercial latex paint is a composed material based on a variety of chemistries, and each latex resin has its own unique properties. Each of these constituent materials plays an important role in the processing and in the final mechanical performance of the product. Although the binder of the paint has the predominant effect on the properties of film formation, the type and quantity of pigments, solvents and additives also have an influence. Latex paints invariably have a number of additives acting as surfactants, coalescing aids, and emulsion stabilizers. After the application of a latex paint to a substrate, the rate at which the water evaporates strongly affects the characteristics of the paint drying process. The Paint2 has vinyl resin in its formulation, which has high electrical polarity. Therefore, this resin tends to bond by hydrogen-bonding attraction with all polar substances present in Paint2, especially with the water [30]. The resultant effect of this bonding is the voids appearing within the paint coating, which induce the expansion effects (Stage I) shown in Figure 7.

In the Stage II, that lasts 220 minutes, a stronger and more complex mechanical deformation was also observed, resulting in a blue wavelength shift of approximately 400 pm. In this stage, stresses in the film deform the particles leading them into an irreversible contact. Finally, in the Stage III, which initiates after 320 minutes from the beginning of the experiment, wavelength shift was found to be almost constant and, therefore, no important mechanical deformation takes place.

At the end of the Paint2 drying process, the compensated FBG4 wavelength suffers a blue shift of 400 pm, corresponding to a decrease of about 417 με in the initial longitudinal strain applied to the FBG4, which relates to a final contraction of approximately 21 μm in the longitudinal film dimension (or equivalently 0.04%). No cracking was observed in the film after the 600 minutes.

Here, the four steps (dry-to-touch, tack-free, dry-to-handle and dry-through) described by Brazilian Technical Standards were also identified and occurs after approximately 65, 95, 100 and 110 minutes, respectively, after the beginning of the experiment for Paint2.

It is important to observe that there is an important difference between paint drying time described by Brazilian Technical Standards and the moment which mechanical deformation can be disregarded. In Figures 6 and 7, the last step dry-through described in the Brazilian Technical Standards NBR 9558 occurs at the beginning of Stage II, and, therefore, it is only a limited human perception of the drying temporal process. At this moment, only approximately 20% of total mechanical deformation took place.

Figure 8 presents the packaged FBG4, FBG5 and FBG6 responses as function of time during the Paint2 drying process mixed with different water concentrations. For the first coating, the drying process for undiluted paint ( 
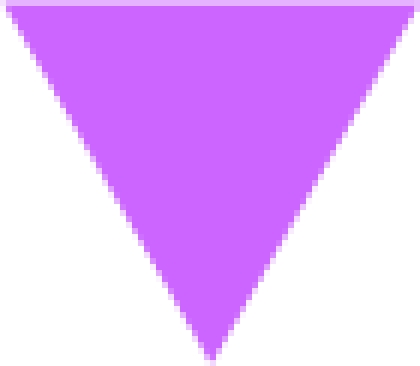
) and for mix of 80% paint to 20% water ( 
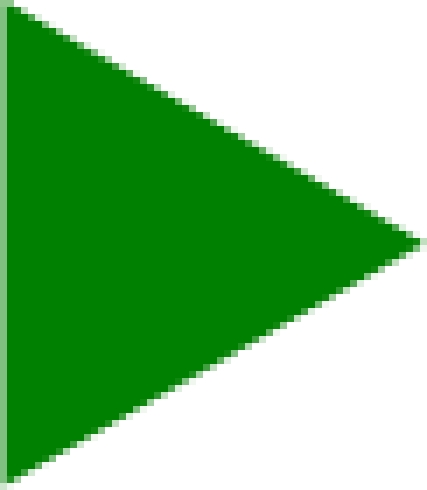
) and for mix of 50% paint to 50% water ( 
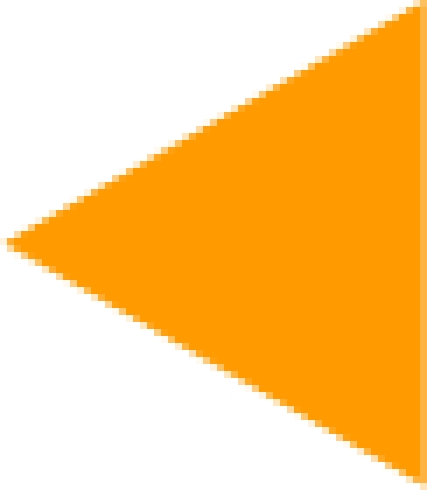
)were investigated. For dilute paints, as in pure paint, three stages with distinct temporal behaviors were also observed. Initially, there are expansion effects denoted by a blue wavelength shift that occurs during, approximately, 30 minutes for paint concentration of 50% and during, approximately, 60 minutes for paint concentration of 80%. The magnitude of expansion effects observed in diluted paint is less important than in undiluted paint: the lower the amount of paint and the smaller the number of voids diminishes, consequently, the magnitude of mechanical expansion.

Following further, there are contraction effects that are indicated by red wavelength shifts. These stages occur in the last 150 minutes and 120 minutes for concentrations of 50% and 80% of paint in the mixture, respectively. For paint concentration of 50%, a total wavelength shift of approximately 100 pm occurs corresponding to a contraction of 104 με. For paint concentration of 80% a total wavelength shift of approximately 200 pm was measured, corresponding to a contraction of 208 με. For both diluted paint, the dry-through time is approximately 170 minutes less than undiluted paint. Therefore, the increase in the water ratio mixed into the paint decreases the mechanical deformation magnitude and also decreases the time in which the mechanical deformation ceases (Stage III). Here, the smaller amount of paint in the samples also plays an important role in the contraction effects during drying process, modifying the time when deformation ceases. As the proportion of water in the paint increases, the rate of water evaporation also increases, decreasing the extent of Stage I. The smaller contraction observed along Stage II for increasing amounts of water reflects a less effective interaction between the paint constituents, once the reduction in the time interval for film formation inhibits the paint particles to effectively get in touch with one another.

## 5. Conclusions

The experimental results obtained in this work show that the strain sensor based on packaged fiber Bragg grating is capable to measure the mechanical deformation during the paint drying process. The knowledge of the mechanical deformation magnitude can lead to a better understanding of the internal stresses involved in the paint drying, supplying information to paint formulators to test film forming properties of their formulation. The potential of this application can be explored and it is clear that it offers an important tool for monitoring paint film formation process.

The stress monitoring during the drying process of two different commercial latex paints showed that three stages (I, II, and III), with distinct mechanical deformation and temporal behaviors, can be identified. In Stage I, which is associated with water evaporation from latex surface, was observed contraction effects in Paint1 and expansion effects in Paint2. In Stage II, which is associated with particles first come into irreversible contact (coalescence effect), for both latex paints were measured mainly contraction effects. Also for both latex paints, the Stage III was characterized by less significant mechanical deformation, indicating that that the drying process is completed. Therefore, we consider that the paint is dry-through only when the mechanical deformation magnitude reaches the Stage III. However, the dry-through time indicated by our optical sensor and by human sense technique based on Brazilian Technical Standards NBR 9558 disagree each other. The human sense technique identifies the end of the drying process before the more strong paint mechanical deformation occurs. Consequently, any technical decision taken based upon the latter technique can lead to large errors on the performance of the coating. The application of optical technique based on fiber Bragg grating for the assessment of the paint drying process can lead to the development of a novel Standard, eliminating both personal and subjective factors.

The monitoring of stress kinetics with optical technique based on fiber Bragg grating allows assessing the influence of the solvent addition in the latex paint drying process. It was observed that the increase of the water ratio mixed into the paint decreases the mechanical deformation magnitude and the paint dry-through time. Apparently, the use of dilute paint should be more advantageous than the use of undiluted paint, because the mechanical deformation magnitude and time in which the mechanical deformation can be disregarded is decreased in comparison with undiluted paint. However, the paint dilution has also negative effects on the film formation properties. The reduction on paint dry-through time caused by the higher evaporation rate of water induces a fragile film formation. For this reason, dilute paint commonly needs multilayer paint coatings to provide optimal corrosion protection or decorative appearance.

## Figures and Tables

**Table 1.** Bragg wavelength position (*λ_B_*), before and after packaging, and the value of its red shift (*δλ*) induced by packaging for each FBG strain sensor.

**Sensor**	***λ_B_* (nm) (Before packaging)**	***λ_B_* (nm) (After packaging)**	***δλ* (nm)**
FBG2	1,540.18	1,540.58	0.40
FBG3	1,541.52	1,542.22	0.70
FBG4	1,540.11	1,540.71	0.60
FBG5	1,540.33	1,540.77	0.44
FBG6	1,539.91	1,540.35	0.44

**Table 2.** Temperature and relative humidity during the experiments of paint drying process.

**Packaged sensor**	**Paint monitored**	**Temperature (°C)**	**Relative humidity (%)**
FBG3	Paint1 (undiluted)	27.4 ± 0.1	51.8 ± 1.0
FBG4	Paint2 (undiluted)	27.6 ± 0.1	56.6 ± 1.0
FBG5	80% Paint2 and 20% water	20.4 ± 0.1	50.5 ± 1.0
FBG6	50% Paint2 and 50% water	25.2 ± 0.1	60.2 ± 1.0
